# Isoliquiritigenin regulates the circ_0002860/miR-431-5p/RAB9A axis to function as a tumor inhibitor in melanoma

**DOI:** 10.1590/1678-9199-JVATITD-2022-0019

**Published:** 2023-03-31

**Authors:** Songjiang Wu, Jian Wang

**Affiliations:** 1 Department of Dermatology, the First Affiliated Hospital of Hengyang Medical College, University of South China, Hengyang, China.; 2 Department of Emergency, the First Affiliated Hospital of Hengyang Medical College, University of South China, Hengyang, China.

**Keywords:** Isoliquiritigenin, circ_0002860, miR-431-5p, RAB9A, Melanoma

## Abstract

**Background:**

Isoliquiritigenin (ISL) presents antitumor effects against melanoma cells. It is known that various circular RNAs (circRNAs) are involved in the development of melanoma. Therefore, the present study aims to investigate the molecular mechanisms of ISL and circ_0002860.

**Methods:**

Circ_0002860, microRNA-431-5p (miR-431-5p) and member RAS oncogene family (RAB9A) were detected through reverse transcription-quantitative polymerase chain reaction (RT-qPCR) assay. Cell viability was examined via cell counting kit-8 assay. The proliferation ability was assessed using colony formation assay. Cell apoptosis and cell cycle were determined by flow cytometry. Transwell assay was used for detection of migration and invasion. Western blot was conducted for protein analysis. Target binding was confirmed via dual-luciferase reporter assay and RNA immunoprecipitation (RIP) assay. *In vivo* research was performed through xenograft tumor assay.

**Results:**

Circ_0002860 was downregulated by ISL in melanoma cells. ISL-induced inhibitory effects on cell proliferation, cell cycle progression, migration and invasion were alleviated by circ_0002860 overexpression. MiR-431-5p was a target of circ_0002860. Circ_0002860 eliminated the ISL-induced tumor inhibition via sponging miR-431-5p in melanoma cells. Circ_0002860 elevated the RAB9A level by targeting miR-431-5p. The function of ISL was related to miR-431-5p/RAB9A axis in melanoma progression. Tumor growth was reduced by ISL *in vivo* through downregulating circ_0002860 to regulate miR-431-5p and RAB9A levels.

**Conclusion:**

The current data indicates that ISL suppressed cell malignant progression of melanoma via targeting the circ_0002860/miR-431-5p/RAB9A pathway.

## Background

Melanoma is one of the most common type of skin cancer arising from pigment-producing melanocytes, with high incidence around the world [1]. The primary cutaneous melanomas can be cured with surgical excision, but those tumors with locoregional metastases have a major risk of recurrence [2, 3]. Isoliquiritigenin (ISL) is an important compound of chalcone, which is isolated from the roots of licorice plants [4]. ISL exhibits various types of biological activities, such as antitumor, anti-oxidative and anti-inflammatory [5]. A recent study has indicated that ISL promotes apoptosis and represses metastasis in melanoma [6]. Xiang *et al*. [7] discovered that ISL reduced cell growth in melanoma via targeting miR-301b/LRIG1 axis. However, the antitumor mechanism of ISL is not fully clear in melanoma.

Circular RNAs (circRNAs) are covalently closed-loop transcripts characterized by regulating gene expression via serving as sponges of microRNAs (miRNAs) [8, 9]. CircRNAs play key roles in the pathogenesis of most skin diseases, including melanoma [10]. Luan *et al*. [11] declared that circRNA_0084043 contributed to the malignant development of melanoma by upregulating Snail through sponging miR-153-3p. The heat map analysis suggested that circ_0002860 was dysregulated with a high level in melanoma tissues [11]. The association of circ_0002860 with the function of ISL in melanoma has never been explored.

MicroRNA-431-5p (miR-431-5p) was identified as an inhibitor in cell growth and migration of melanoma via targeting NOTCH2 [12]. Circ_0001591 facilitated melanoma cell proliferation and metastasis through miR-431-5p sponging function to mediate ROCK1 level [13]. Member RAS oncogene family (RAB9A) exerted the oncogenic function in tumorigenicity and development of melanoma [14, 15]. Zhang *et al.* [16] elucidated that circ_0013359 promoted the melanoma progression through the miR-136-5p-mediated RAB9A upregulation. Whether circ_0002860 can induce the sponge effect to regulate RAB9A remains unknown in melanoma cells.

This research was conducted to investigate the relation of the tumor-inhibitory role of ISL with circ_0002860. The interaction between circ_0002860 or RAB9A and miR-431-5p was explored. The underlying mechanism of ISL with circ_0002860/miR-431-5p/RAB9A was unraveled in melanoma.

## Methods

### Human samples and cell lines

The melanoma tissues (n = 50) and normal paracancerous tissues (n = 50) were acquired from the First Affiliated Hospital of Hengyang Medical College, University of South China. The written informed consent files were provided by all melanoma patients. This study has approved by the Ethics Committee of the First Affiliated Hospital of Hengyang Medical College, University of South China (approval number: HN20190822). The collected samples were stored in liquid nitrogen until the extraction of RNA or protein.

A875 and SK-MEL-1 cell lines (Jennio Biotech, Guangzhou, China) were used for the research of m,anoma, and they were maintained in Dulbecco’s modified Eagle’s medium (DMEM; 11966025, Gibco) with 10% fetal bovine serum (FBS; 10099141C, Gibco). Human epidermal melanocytes, neonatal, lightly pigmented donor (HEMn-LP; Gibco. Carlsbad, CA, USA) were incubated with M254 medium (M254500, Gibco) including Human Melanocyte Growth Supplement (S0025, Gibco). All these cells were cultivated in the 5% CO_2_ incubator at 37 °C.

### Cell treatment and transfection

For cell treatment, A875 and SK-MEL-1 cells were exposed to ISI (I3766, Sigma, St. Louis, MO, USA) with increasing concentrations (10-80 μg/mL) for 24 h. For cell transfection, 1 × 10^4^ cells were planted into the 96-well plates and added with vectors or oligonucleotides via Lipofectamine™ 3000 kit (L3000001, Invitrogen, Carlsbad, CA, USA). The pcD5-ciR vector (GENESEED, Guangzhou, China) was cloned with circ_0002860 sequence for overexpression of circ_0002860 (oe-circ_0002860). The miR-431-5p mimic, miR-NC mimic, miR-431-5p inhibitor (anti-miR-431-5p), anti-miR-NC, small interfering RNA of RAB9A (si-RAB9A) and si-NC were commercially obtained from GenePharma (Shanghai, China).

### Reverse transcription-quantitative polymerase chain reaction (RT-qPCR) assay

RNA samples from tissues and cells were extracted via TRI Reagent (T9424, Sigma), followed by cDNA synthesis through ReverTra Ace® qPCR RT Kit (FSQ-101, Toyobo, Kita-Ku, Osaka, Japan) and level detection using SYBR^®^ Green Realtime PCR Master Mix (QPK-201, Toyobo). Then the levels were standardized to β-actin and U6, and the relative analysis was implemented using the 2^-∆∆Ct^ method [17]. Additionally, circ_0002860 stability was detected via RT-qPCR. Cells were incubated with 3 mg/mL Actinomycin D (Sigma) or total RNA was treated with 3 U/μg RNase R (GENESEED), then circ_0002860 and linear CFLAR levels were quantified by RT-qPCR. All primer sequences were provided in Table 1.

**Table 1.** Primer sequences used for RT-qPCR.

Name	Primer sequences (5’-3’)	
circ_0002860	Forward	GAGCAAGCCCCTAGGAATCTG
	Reverse	CAGTCAACAGAAAGCCAGCAG
CFLAR	Forward	CCTCACCGACGAGTCTCAAC
	Reverse	GCGCTTCTCTCCTACACCTC
miR-1197	Forward	GTATGATAGGACACATGGT
	Reverse	CTCAACTGGTGTCGTGGAG
miR-421	Forward	GTATGAATCAACAGACATTAA
	Reverse	CTCAACTGGTGTCGTGGAG
miR-431-5p	Forward	TCGGCAGGTGTCTTGCAGGCC
	Reverse	CTCAACTGGTGTCGTGGAG
miR-576-5p	Forward	TCGGCAGGATTCTAATTTCTCC
	Reverse	CTCAACTGGTGTCGTGGAG
RAB9A	Forward	CTCTCTGTCCTCATTGCGCC
	Reverse	ACCCTCCTAGGGTTGTTGAGA
β-actin	Forward	GGGAAATCGTGCGTGACATTAAG
	Reverse	GTCAGGCAGCTCGTAGCTCT
U6	Forward	CTCGCTTCGGCAGCACA
	Reverse	AACGCTTCACGAATTTGCGT

### Cell counting kit-8 (CCK-8) assay

After ISL treatment and cell transfection for 24 h, melanoma cells were incubated with 10 μL/well CCK-8 solution (KGA317, KeyGen, Nanjing, China) at 37 °C. After 2 h, cell absorbance of 450 nm was tested through the microplate reader and the percentage of viable cells in total cells was calculated.

### Colony formation assay

Five-hundred cells were added into the 12-well plates. Cells were cultured for 14 days, then the white colonies were fixated with 4% paraformaldehyde (Sigma) for 10 min and stained with 0.1% crystal violet (Sigma) for 15 min. Cell colonies were photographed using a digital camera and counted by Image J software (NIH, Bethesda, MD, USA).

### Flow cytometry

Cell apoptosis was evaluated via apoptosis kit (KGA106, KeyGen). A875 and SK-MEL-1 cells (5 × 10^5^) in 200 µL binding buffer reacted with 10 µL annexin V-fluorescein isothiocyanate (annexin V-FITC) and 10 µL propidium iodide (PI). After co-incubation for 20 min, cell status was detected through the flow cytometer (BD Biosciences, San Diego, CA, USA) and apoptosis rate was expressed as the ratio of apoptotic cells in total cells.

Cell cycle was determined by Cell Cycle Detection Kit (KGA511, KeyGen). A875 and SK-MEL-1 cells (1 × 10^6^) were centrifuged at 2000 rpm for 5 min and cell pellets were fastened with 500 µL 70% cold ethanol (Sigma) at 4 °C overnight. Then, cells were washed with 200 µL phosphate buffer solution (Gibco) and added with 500 µL PI/RNase A working solution for 45 min. The red fluorescence at excitation wavelength 488 nm was examined under the flow cytometer (BD Biosciences), and DNA content was analyzed in different phases (G1, S, G2, M).

### Transwell assay

Transwell chamber (Corning Inc., Corning, NY, USA) was used for migration assay and chamber enveloped with matrigel (Corning Inc.) was applied to measure cell invasion. The lower chamber was added with 500 µL cell medium and the upper chamber was seeded with 1 × 10^5^ cells. After incubation for 24 h, the moved cells were fixated with 4% paraformaldehyde (Sigma) and dyed with 0.1% crystal violet (Sigma) for 20 min. Cell images were acquired at 100 × magnification and cells were counted through the inverted microscope (Olympus, Tokyo, Japan).

### Western blot

Total proteins were isolated by whole cell lysis assay kit (KGP250, KeyGen) and the concentrations were tested using BCA protein quantitation assay kit (KGP902, KeyGen), according to the manufacturer’s guidelines. The protein level was measured by western blot assay as previously depicted [18]. All antibodies were bought from Cell Signaling Technology (CST, Boston, MA, USA). The antibody information was exhibited as follows: anti-E-cadherin (#3195, 1:1000), anti-N-cadherin (#13116, 1:1000), anti-vimentin (#5471, 1:1000), anti-β-actin (#4970, 1:1000), anti-RAB9A (#5118, 1:1000), anti-rabbit IgG, HRP-linked antibody (#7074, 1:3000). SignalFire™ Plus ECL Reagent (CST) was employed for protein presentation and Image J software (NIH) was used for grey level analysis.

### Dual-luciferase reporter assay

Circ_0002860 and RAB9A 3’UTR sequences contained the miR-431-5p binding sites, which were considered as the wild-type (WT) sequences. The sequences containing the mutated sites for miR-431-5p were considered as the mutant-type (MUT) sequences. Luciferase reporters of circ_0002860 (circ_0002860 WT, circ_0002860 MUT) and RAB9A (RAB9A 3’UTR WT, RAB9A 3’UTR MUT) were constructed using the pmirGLO plasmid (Promega, Madison, WI, USA). A875 and SK-MEL-1 cells were transfected with each reporter and miR-NC mimic or miR-431-5p mimic at 37 °C for 48 h. Subsequently, luciferase activity of each well was determined via the Dual-Luciferase Reporter Detection System (Promega).

### RNA immunoprecipitation (RIP) assay

Magna RIP RNA-Binding Protein Immunoprecipitation Kit (RIP-12RXN, Sigma-Aldrich) was used for binding analysis between circ_0002860 and miR-431-5p. A875 and SK-MEL-1 cells were incubated with the antibody-conjugated magnetic beads at 4 °C overnight. Anti-immunoglobulin G (anti-IgG) was used as the negative control group of anti-argonaute-2 (anti-Ago2). Input group without incubation of magnetic beads acted as the positive group. Total RNA was extracted from the beads, followed by detecting the levels of circ_0002860 and miR-431-5p.

### Animal experiment

 After transfection of vector or oe-circ_0002860 for 48 h, A875 cells (2 × 10^6^) were subcutaneously injected into BALB/c male nude mice (Vital River Laboratory Animal Technology Co., Ltd., Beijing, China). When tumors reached 50-100 mm^3^, mice were intraperitoneally injected with 20 mg/kg ISL or PBS every two days. There were 5 mice in PBS+vector, ISL+vector or ISL+oe-circ_0002860 group. Tumor volume (length × width^2^ × 0.5) was examined every week, and mice were euthanatized through flowing CO_2_ after four weeks. Tumors were excised from mice and weighed. The gene levels were determined via RT-qPCR and western blot. The protein level of Ki67 (CST, #9027) was measured using Immunohistochemistry (IHC) assay [19]. All procedures for animals were ratified by the Animal Ethical Committee of the First Affiliated Hospital of Hengyang Medical College, University of South China (approval number: HN20190822).

### Statistical analysis

The experiments were performed with three replicates, then data were revealed as the mean ± standard deviation and analyzed via SPSS 22.0 (SPSS Inc., Chicago, IL, USA). The group difference was calculated using Student’s *t*-test or analysis of variance (ANOVA) followed by Tukey’s test, and *p* < 0.05 showed a significant difference.

## Results

### ISL downregulated the level of circ_0002860 in melanoma cells

**Figure 1.**
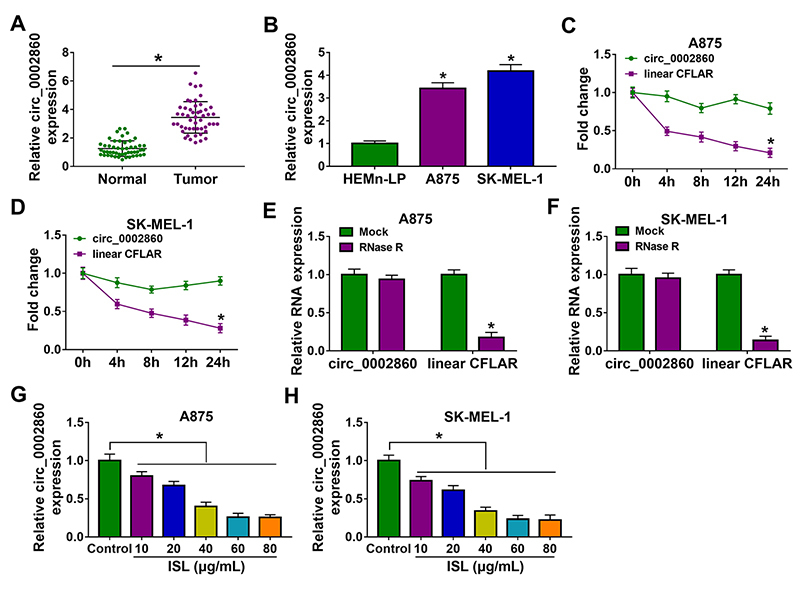
ISL downregulated the level of circ_0002860 in melanoma cells. RT-qPCR was used for circ_0002860 level detection in melanoma **(A)** tissues and **(B)** cells. **(C, D)** Circ_0002860 and CFLAR levels were determined after A875 and SK-MEL-1 cells were incubated with actinomycin D for 0 h, 4 h, 8 h, 12 h and 24 h. **(E, F)** The quantification of circ_0002860 and CFLAR was performed by RT-qPCR after total RNA was treated with RNase R for 1 h. **(G, H)** The circ_0002860 expression was assayed using RT-qPCR after treatment of ISL (10 µg/mL, 20 µg/mL, 40 µg/mL, 60 µg/mL, 80 µg/mL) in A875 and SK-MEL-1 cells. *p < 0.05.

RT-qPCR data demonstrated that circ_0002860 was highly expressed in melanoma tissues (Figure 1A) and A875/SK-MEL-1 cells (Figure 1B), relative to normal tissues and HEMn-LP cells. The linear CFLAR expression was markedly reduced while circ_0002860 was more resistant to the treatment of Actinomycin D (Figures 1C, 1D) and RNase R (Figures 1E, 1F), suggesting the high stability of circ_0002860 in A875 and SK-MEL-1 cells. The level of circ_0002860 was significantly inhibited by ISL with different concentrations, compared to the control group (Figures 1G, 1H). Circ_0002860 might be related to the function of ISL in melanoma.

### ISL impeded proliferation, cell cycle progression, migration and invasion of melanoma cells via inhibiting circ_0002860

**Figure 2.**
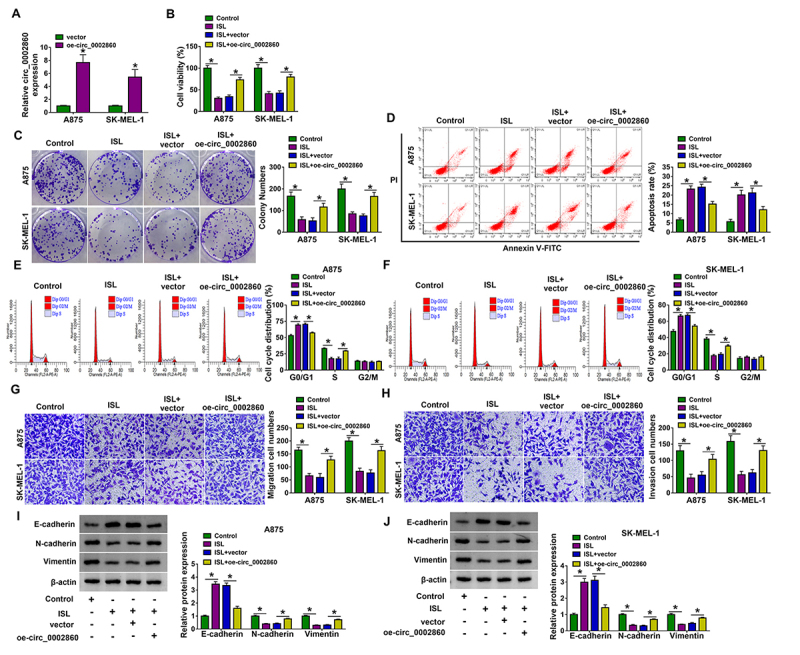
ISL impeded proliferation, cell cycle progression, migration and invasion of melanoma cells via inhibiting circ_0002860. **(A)** The expression analysis of circ_0002860 was performed via RT-qPCR in vector or oe-circ_0002860 transfected A875 and SK-MEL-1 cells. A875 and SK-MEL-1 cells were treated with control, ISL (60 µg/mL), ISL+vector, ISL+oe-circ_0002860. **(B)** Cell viability examination was performed via CCK-8 assay. **(C)** Cell proliferation detection was conducted by colony formation assay. The determination of **(D)** cell apoptosis and **(E, F)** cell cycle was performed by flow cytometry. The evaluation of **(G)** migration and **(H)** invasion was conducted via transwell assay. **(I, J)** The measurement of EMT-related proteins was performed via western blot. *p < 0.05.

The overexpression of circ_0002860 was achieved by transfection of oe-circ_0002860, and the efficiency was great contrasted with vector transfection in A875 and SK-MEL-1 cells (Figure 2A). ISL suppressed cell viability in CCK-8 assay (Figure 2B) and cell proliferation in colony formation assay (Figure 2C), but oe-circ_0002860 eliminated these influences. Flow cytometry manifested that circ_0002860 overexpression attenuated the promotion of cell apoptosis (Figure 2D) and inhibition of cell cycle progression (Figures 2E, 2F) caused by ISL. By performing the transwell assay, cell migration and invasion abilities were shown to be enhanced by transfection of oe-circ_0002860 in ISL-treated A875 and SK-MEL-1 cells (Figures 2G, 2H). The protein analysis of epithelial-mesenchymal transition (EMT) indicated that ISL-induced E-cadherin upregulation and N-cadherin or Vimentin downregulation were relieved after circ_0002860 was overexpressed (Figures 2I, 2J). Altogether, ISL inhibited the progression of melanoma cells by downregulating circ_0002860.

### Circ_0002860 induced the sponge effect on miR-431-5p

**Figure 3.**
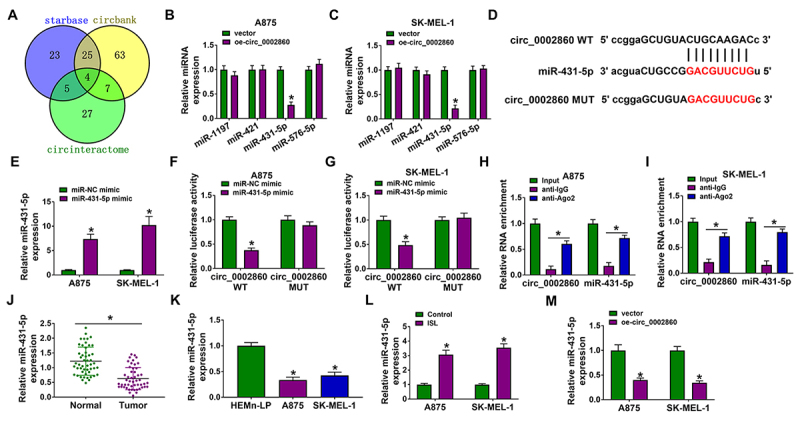
Circ_0002860 induced the sponge effect on miR-431-5p. **(A)** Venn Diagram was applied to select the mutual miRNAs by starbase, circbank and circinteractome. **(B, C)** The miR-1197, miR-421, miR-431-5p and miR-576-5p levels were examined by RT-qPCR after A875 and SK-MEL-1 cells were transfected with vector or oe-circ_0002860. **(D)** The binding region between circ_0002860 and miR-431-5p in starbase. **(E)** The transfection efficiency of miR-431-5p mimic was assessed using RT-qPCR. **(F-G)** Dual-luciferase reporter assay and **(H-I)** RIP assay were used to analyze the combination of circ_0002860 and miR-431-5p. The miR-431-5p quantification was performed using RT-qPCR in **(J)** melanoma samples and **(K)** cells. **(L)** The level of miR-431-5p was measured by RT-qPCR in 60 µg/mL ISL-treated A875 and Sk-MEL-1 cells. **(M)** The miR-431-5p expression was detected by RT-qPCR after transfection with vector or oe-circ_0002860. *p < 0.05.

The miRNA targets for circ_0002860 were predicted by starbase (http://starbase.sysu.edu.cn), circbank (http://www.circbank.cn/index.html) and circinteractome (https://circinteractome.nia.nih.gov/), then Venn diagram exhibited that 4 miRNA targets (miR-1197, miR-421, miR-431-5p, miR-576-5p) might be targets of circ_0002860 (Figure 3A). The expression detection showed that miR-431-5p was the only miRNA downregulated by circ_0002860 in A875 and SK-MEL-1 cells (Figures 3B, 3C). The binding sites between miR-431-5p and circ_0002860 were displayed in starbase (http://starbase.sysu.edu.cn) (Figure 3D). The miR-431-5p level was higher in miR-431-5p mimic group than that in miR-NC mimic group of A875 and SK-MEL-1 cells (Figure 3E). With the overexpression of miR-431-5p, the luciferase activity was repressed in circ_0002860 WT group but nor circ_0002860 MUT group (Figures 3F, 3G). Circ_0002860 and miR-431-5p were enriched in anti-Ago2 group compared to anti-IgG group (Figures 3H, 3I), which also affirmed the interaction between circ_0002860 and miR-431-5p. The downregulation of miR-431-5p was detected in melanoma tissues (Figure 3J) and cells (Figure 3K) relative to the normal controls. Additionally, miR-431-5p expression was elevated following treatment of ISL in A875 and SK-MEL-1 cells (Figure 3L). The miR-431-5p level was reduced after overexpression of circ_0002860 in A875 and SK-MEL-1 cells (Figure 3M). These results could validate that circ_0002860 acted as a miR-431-5p sponge.

### ISL inhibited the melanoma progression by targeting the circ_0002860/miR-431-5p axis

**Figure 4.**
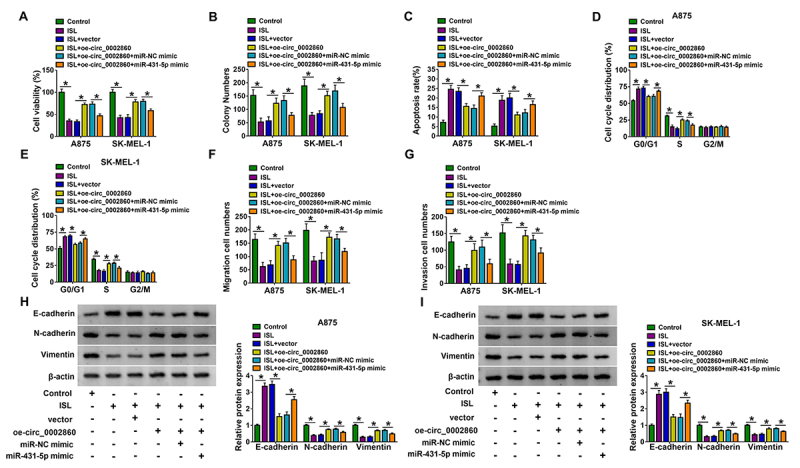
ISL inhibited the melanoma progression by targeting the circ_0002860/miR-431-5p axis. A875 and SK-MEL-1 cells were treated with control, ISL (60 µg/mL), ISL+vector, ISL+oe-circ_0002860, ISL+oe-circ_0002860+miR-NC mimic, ISL+oe-circ_0002860+miR-431-5p mimic. **(A)** Cell viability and **(B)** proliferation were detected by CCK-8 assay and colony formation assay. **(C)** Cell apoptosis and **(E, F)** cell cycle were assessed by flow cytometry. **(F)** The migration and **(G)** invasion abilities were determined by transwell assay. **(H, I)** EMT markers were examined by western blot. *p < 0.05.

Furthermore, the relation between circ_0002860/miR-431-5p axis and ISL was researched in A875 and SK-MEL-1 cells. The circ_0002860-mediated cell viability enhancement (Fig. 4A), proliferation promotion (Figure 4B), apoptosis inhibition (Figure 4C) and cell cycle acceleration (Figures 4D, 4E) in ISL-treated cells were all reversed by miR-431-5p mimic. As the results of miR-431-5p overexpression, the promoting effects of or-circ_0002860 on cell migration/invasion (Figures 4F, 4G) and EMT process (Figures 4H, 4-I) were counteracted in part. Thus, the regulation of circ_0002860 was partly achieved by sponging miR-431-5p in ISL-treated melanoma cells.

### Circ_0002860 sponged miR-431-5p to increase the RAB9A level in melanoma cells

**Figure 5.**
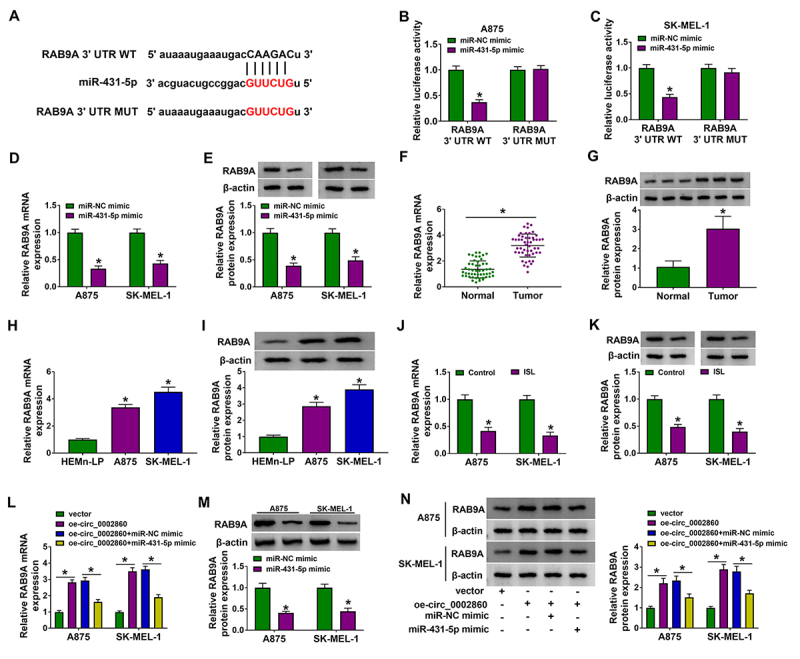
Circ_0002860 sponged miR-431-5p to increase the RAB9A level in melanoma cells. **(A)** Starbase exhibited the binding site between RAB9A 3’UTR and miR-431-5p. **(B, C)** The binding between miR-431-5p and RAB9A was identified by dual-luciferase reporter assay. **(D, E)** The mRNA and protein levels of RAB9A were measured via RT-qPCR and western blot after transfection with miR-NC mimic or miR-431-5p mimic. RAB9A quantification was performed using RT-qPCR and western blot in **(F, G)** melanoma tissues and **(H, I)** cells. **(J, K)** The effect of 60 µg/mL ISL on RAB9A expression was evaluated via RT-qPCR and western blot. **(L)** RT-qPCR was applied to detect the RAB9A mRNA level in vector, oe-circ_0002860, oe-circ_0002860+miR-NC mimic and oe-circ_0002860+miR-431-5p mimic groups. **(M)** RAB9A protein level was detected by western blot after transfection of miR-NC mimic or miR-431-5p mimic. **(N)** RAB9A protein expression was determined by western blot after transfection as Figure 5L. *p < 0.05.

The prediction of starbase showed that RAB9A 3’UTR sequence had the miR-431-5p binding sites (Figure 5A). Dual-luciferase reporter assay demonstrated that luciferase activity was reduced after co-transfection of RAB9A 3’UTR WT and miR-431-5p mimic rather than RAB9A 3’UTR MUT and miR-431-5p mimic (Figures 5B, 5C). RAB9A mRNA and protein levels were obviously inhibited by transfection of miR-431-5p mimic compared with transfection of miR-NC mimic (Figures 5D, 5E). Through the detection of RT-qPCR and western blot, RAB9A was found to be upregulated in melanoma tissues (Figures 5F, 5G) and cells (Figures 5H, 5I). Then, we discovered that ISL resulted in the mRNA and protein downregulation of RAB9A in A875 and SK-MEL-1 cells (Figures 5J, 5K). Moreover, circ_0002860 overexpression promoted the mRNA expression of RAB9A while this change was mitigated by miR-431-5p (Figure 5L). Overexpression of miR-431-5p downregulated RAB9A protein expression in A875 and SK-MEL-1 cells (Figure 5M). RAB9A protein upregulation induced by oe-circ_0002860 was also recovered by miR-431-5p overexpression (Figure 5N). Overall, circ_0002860 could regulate RAB9A via targeting miR-431-5p.

### The miR-431-5p inhibition counterbalanced the ISL-induced antitumor function by upregulating RAB9A in melanoma cells

**Figure 6.**
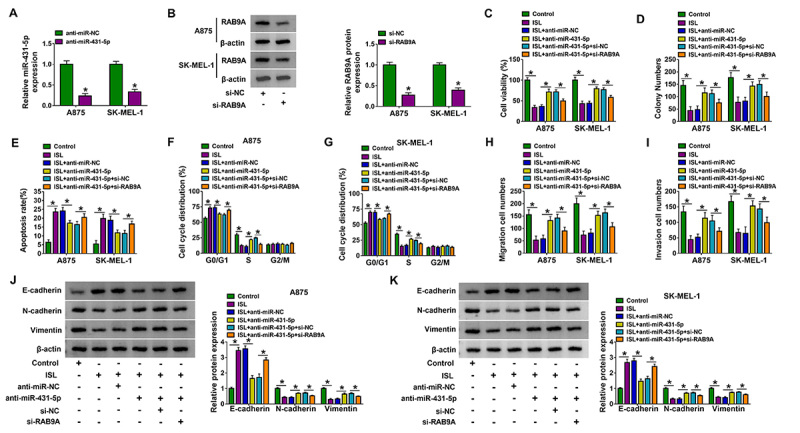
The miR-431-5p inhibition counterbalanced the ISL-induced antitumor function by upregulating RAB9A in melanoma cells. The inhibitory efficacies of **(A)** anti-miR-431-5p and **(B)** si-RAB9A were analyzed through RT-qPCR and western blot, respectively. The control, ISL (60 µg/mL), ISL+anti-miR-NC, ISL+anti-miR-431-5p, ISL+anti-miR-431-5p+si-NC and ISL+anti-miR-431-5p+si-RAB9A groups were designed in A875 and SK-MEL-1 cells. CCK-8 assay and colony formation assay were conducted to determine **(C)** cell viability and **(D)** proliferation. Flow cytometry was conducted to examine **(E)** cell apoptosis and **(F, G)** cell cycle progression. Transwell assay was implemented to assess **(H)** cell migration and **(I)** invasion. **(J-K)** Western blot was performed to analyze the protein levels of EMT-associated markers. *p < 0.05.

The effects of miR-431-5p and RAB9A were explored in ISL-treated cells. The data of RT-qPCR and western blot indicated that expression inhibition of miR-431-5p and RAB9A mediated by anti-miR-431-5p and si-RAB9A was conspicuous in A875 and SK-MEL-1 cells (Figures 6A, 6B). ISL-induced cell viability and proliferation suppression (Figures 6C, 6D), apoptosis acceleration (Figure 6E) and cell cycle arrest (Figures 6F, 6G) were alleviated by anti-miR-431-5p, which was then abolished by knockdown of RAB9A. The introduction of si-RAB9A also abrogated the promoting influences of anti-miR-431-5p on migration (Figure 6H), invasion (Figure 6I) and EMT (Figures 6J, 6K) after A875 and SK-MEL-1 cells were exposed to ISL. The miR-431-5p/RAB9A axis was partly responsible for the antitumor response of ISL in melanoma cells.

### ISL reduced tumor growth of melanoma *in vivo* by targeting circ_0002860 to regulate the miR-431-5p/RAB9A axis

**Figure 7.**
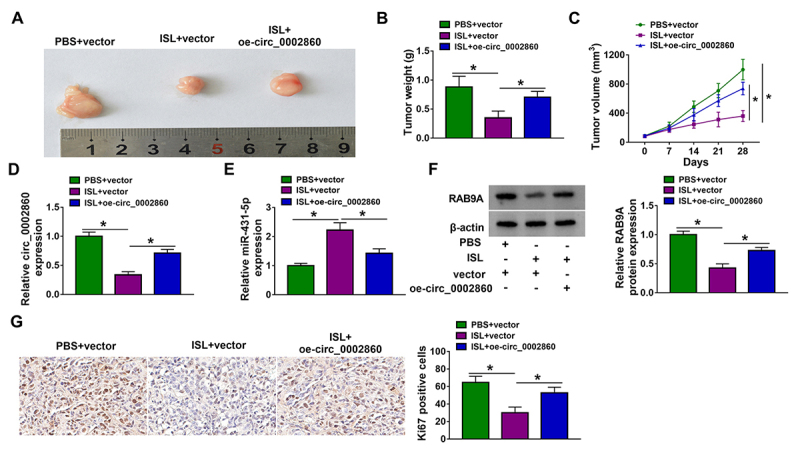
ISL reduced tumor growth of melanoma *in vivo* by targeting circ_0002860 to regulate the miR-431-5p/RAB9A axis. **(A)** Tumor pictures in xenograft models of PBS+vector, ISL+vector and ISL+oe-circ_0002860 groups. **(B)** Tumor weight and **(C)** volume of each group. RT-qPCR was employed for detecting the levels of **(D)** circ_0002860 and **(E)** miR-431-5p in tissues. **(F)** RAB9A and **(G)** Ki67 protein levels were measured via western blot and IHC assay, respectively.

To investigate the association of ISL with circ_0002860, xenograft tumor models were constructed in mice. Tumor images were shown in Figure 7A. The weight and volume of tumors were inhibited in ISL+vector group relative to PBS+vector group, then ISL-induced tumor growth inhibition was offset by circ_0002860 overexpression (Figures 7B, 7C). RT-qPCR manifested that circ_0002860 downregulation (Figure 7D) and miR-431-5p upregulation (Figure 7E) were caused by ISL treatment, while these effects were abated by circ_0002860 level upregulation in mice. Also, the protein levels of RAB9A (Figure 7F) and Ki67 (Figure 7G) were enhanced in ISL+oe-circ_0002860 group contrasted with ISL+vector group. Taken together, tumor growth was suppressed by ISL via mediating the circ_0002860/miR-431-5p/RAB9A axis.

## Discussion

ISL has been affirmed to inhibit the progression of human malignancies. For instance, cell proliferation was reduced and apoptosis was enhanced by ISL in nasopharyngeal carcinoma via affecting the miR-32/LATS2/Wnt axis [20]. ISL impeded breast cancer cell proliferation and metastasis via mediating miR-374a/PTEN/Akt network [21]. Huang *et al*. [22] stated that ISL downregulated cyclinD1 to inactive PI3K/AKT pathway, thus repressing oncogenesis and migration of hepatocellular carcinoma cells. The inhibitory effects of ISL on melanoma cell growth and cell cycle progression, as well as the promoting influence on apoptosis, were affirmed in melanoma cells. As for cell metastasis, ISL has resulted in inhibition of cell migration and invasion. In addition, EMT-related protein detection also demonstrated that ISL reduced the motility ability of melanoma cells. Collectively, our results confirmed that ISL acted as a tumor inhibitor in melanoma.

Increasing circRNAs were differentially expressed and participated in the malignant development of melanoma. Circ_0020710 was upregulated in melanoma and promoted tumor progression *in vitro* [23]. CircRNA_0016418 and circZNF609 accelerated the biological behaviors including migration, invasion and EMT in melanoma cells [24, 25]. Herein, circ_0002860 level was shown to be significantly elevated in melanoma samples and cells. Interestingly, circ_0002860 was downregulated in ISL-treated melanoma cells and circ_0002860 upregulation attenuated the ISL-induced effects on cell processes. Therefore, the antitumor role of ISL in melanoma was associated with the inhibition of circ_0002860.

CircRNA/miRNA/mRNA axis has been reported in different types of human tumors. CircRNA_102209 facilitated cell growth and development in colorectal cancer via targeting miR-761 to increase the RIN1 expression [26]. CircRNA LPAR3 could interact with miR-198/MET axis to enhance the metastatic capacity of esophageal cancer cells [27]. Circ_0074026 upregulated the ERBB4 level by absorbing miR-1304, thereby promoting carcinogenesis in glioma cells [28]. The current data suggested that circ_0002860 was a miRNA sponge of miR-431-5p and the downstream target RAB9A was regulated by circ_0002860 through mediating miR-431-5p in melanoma cells. Furthermore, cell assays manifested that ISL inhibited the melanoma progression via regulating the circ_0002860/miR-431-5p axis and miR-431-5p/RAB9A axis. Hence, ISL was considered to act as a tumor repressor by targeting circ_0002860 to modulate miR-431-5p/RAB9A axis. Meanwhile, animal assay validated that circ_0002860 could mitigate the ISL-induced tumor growth suppression by the regulation of miR-431-5p and RAB9A levels.

For the first time, the functional mechanism of ISL with circRNA/miRNA/mRNA axis was disclosed in melanoma. Nevertheless, this study still has some limitations. For example, ISL has been indicated to be associated with signaling pathways in tumors. Exploring the downstream pathways of circ_0002860/miR-431-5p/RAB9A axis will be beneficial for the understanding of ISL-induced antitumor function. In addition, the regulation of circ_0002860 was partly achieved by miR-431-5p/RAB9A axis in alleviating the effects of ISL. Other miRNA/mRNA networks remain to be explored for circ_0002860.

## Conclusion

**Figure 8.**
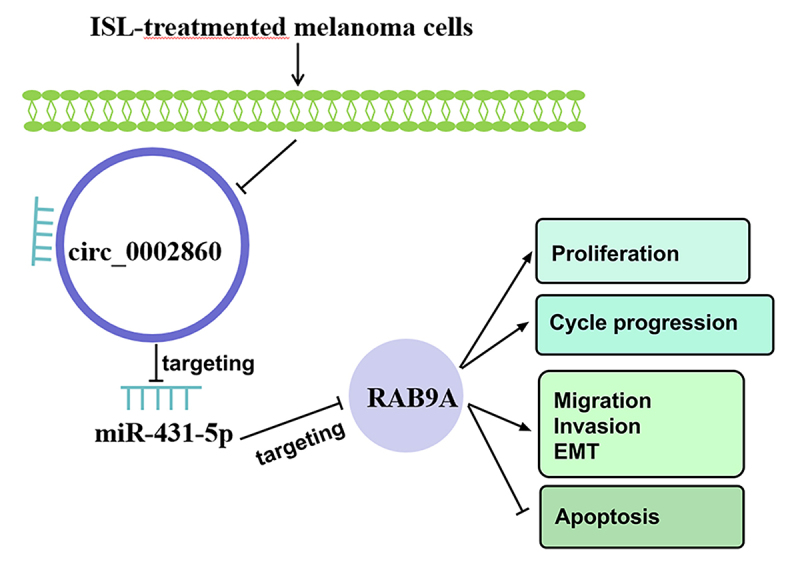
The mechanical diagram of ISL in melanoma cells.

In conclusion, ISL inhibited cell progression (proliferation, cell cycle progression, migration, invasion and EMT) and promoted apoptosis of melanoma via mediating the circ_0002860/miR-431-5p/RAB9A axis (Figure 8). Circ_0002860 might be used as a molecular target for ISL treatment in melanoma.
